# The synergetic effect of pulp chamber extension depth and occlusal thickness on stress distribution of molar endocrowns: a 3-dimensional finite element analysis

**DOI:** 10.1007/s10856-022-06677-0

**Published:** 2022-06-20

**Authors:** Yuejiao Zhang, Hongbin Lai, Qingzhen Meng, Qimei Gong, Zhongchun Tong

**Affiliations:** 1 Department of Operative Dentistry and Endodontics, Hospital of Stomatology, Sun Yat-sen University, Guangzhou, Guangdong China; 2 Guangdong Provincial Key Laboratory of Stomatology, Sun Yat-sen University, Guangzhou, Guangdong China

## Abstract

The aim of this study was to evaluate the effects of butt margin, occlusal thickness and pulp chamber extension depth on stress distributions on mandibular molar endodontically treated teeth (ETT) with EMAX endocrown restoration using 3-dimensional finite element analysis (FEA). The FEA models of endocrown with flat surface or curve surface of butt margin were firstly evaluated stress distributions, and then 9 FEA models of endocrown with 1-, 2- or 3-mm pulp chamber extension depth and 1-, 2- or 3-mm occlusal thickness were generated using curve surface of butt margin. In all of FEA models, a 200 N of vertical load or horizontal load was applied, and the von Mises stress (VMS) were evaluated. The results showed that curve surface of butt margin offered more adhesive area of enamel, though VMS on the prepared teeth was similar in flat surface and curve surface models. In 9 endocrown models, 2-mm occlusal thickness showed the lowest VMS on restorations, teeth tissue and root furcations, and 2-mm extension depth displayed the lowest VMS on root furcations under vertical load. Also, 2-mm extension depth exhibited the lowest VMS on restorations and teeth tissue under horizontal load. Within the limitations of this FEA study, the results of this study could be used as an aid for dentists to better devise endocrown restorations.

**Figure:**
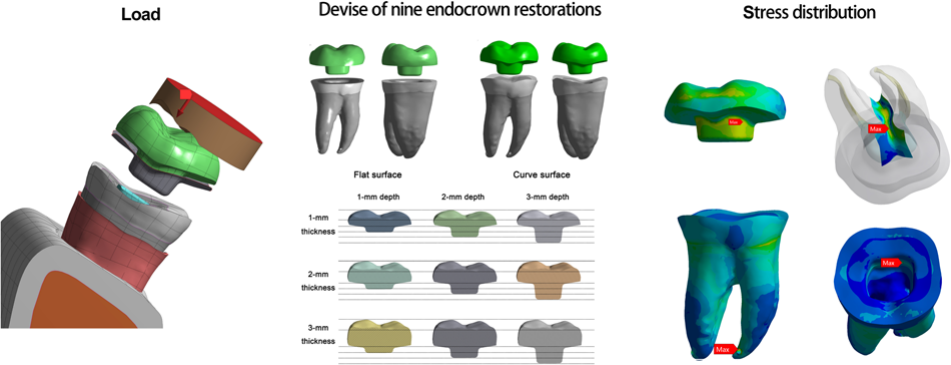
Graphical abstract

Graphical abstract

## Introduction

Rehabilitation of endodontically treated teeth (ETT) with extensive damage is a challenge in restoration dentistry. The most common and accepted approach for these ETT involves a full crown prosthesis by post extension in the canal and a core build-up. However, intracanal posts weaken the tooth structure and increase the risk of root fracture accompanied by pile path preparation [1, 2]. Furthermore, ETT with vertical bite height loss or inadequate clinical crown length is common in clinic, and it’s difficult to establish an adequate ferrule in full crown preparation and achieve a good retention and stability.

With the progress in adhesive dentistry and emphasis on minimally invasive principles, endocrown has been proposed to apply in restoration of ETT with serious defects [2, 3]. Endocrown consists of the entire core and crown that are retained by macro-mechanical retention provided by the internal wall of the pulp chamber and micro-retention/chemical bonding with adhesive cementation. Endocrown requires neither a ferrule nor a post to increase the retention, and is a conservative treatment modality for the restoration of ETT. A few in vitro researches indicated that the teeth with endocrown restoration had similar or higher fracture resistance than the teeth with post-core and conventional crown restoration, and endocrown had similar to or higher success rate than conventional crown in the clinical trials [2–7]. Therefore, endocrown can be considered as an alternative to conventional post-core and crown restoration. Like conventional ceramic crown, the endocrown is usually fabricated by milling a ceramic block using computer-aided techniques or by molding ceramic materials [8, 9].

The design of endocrown restoration is still in dispute in the restoration of ETT with the serious lesion. How to better devise endocrown become the emphasis for dentists. The mechanical behavior of endocrown restoration is influenced by the several factors, such as the marginal forms, abduction angle, pulp chamber extension depth, occlusal thickness and wall thickness of pulp chamber [10–14]. Butt margins and pulp chamber extension depth are two routinely studied factors in evaluating the fracture resistance of endocrown restoration. The flat preparation for butt margin was mostly employed in the researches on endocrown design, and often compared with the anatomic and ferrule preparation [10, 11, 15]. The preparation for pulp chamber extension depth exists divergence in a series of researches. Dartora et al. considered that the greater extension depth of pulp chamber offered better retention and mechanical performance [14]. The other studies showed that the greater extension depth of pulp chamber would result in catastrophic fracture [8, 16, 17]. Additionally, occlusal thickness of endocrown was also considered a key factor to influence fracture resistance in some researches [10, 18, 19].

Occlusal thickness of endocrown depends on lesion of teeth, preparation of teeth, vertical bite height and clinical crown length, and thereby occlusal thickness of endocrown is variable in clinic. It is necessary for dentists to consider the effect of restoration thickness on pulp chamber extension depth in endocrown restoration. Furthermore, whether flat preparation for butt margin is reasonable for endocrown restoration is still questionable due to anatomic occlusal morphology of teeth crown. Therefore, in the light of the factors, we set up the models of mandibular molar ETT with endocrown restoration fabricated by lithium disilicate glass-ceramic using 3-dimensional finite element analysis (FEA), and aimed to (1) firstly compare the stress distribution of endocrown restoration with flat and curve surface butt margin; (2) then evaluate stress distributions on ETT restored with nine types of endocrown with 1-mm, 2-mm, or 3-mm pulp chamber extension depth in combination with 1-mm, 2-mm, or 3-mm occlusal thickness. The null hypotheses of this study were that (1) two types of butt margin would not affect stress distributions on endocrown restorations; (2) Pulp chamber extension depth and occlusal thickness of endocrowns had no influence on stress distributions on restorations and teeth.

## Methods and materials

### Generation of the geometric model

A 3-D geometric model of an intact mandibular molar was obtained from microcomputed tomography (uCT50, Switzerland) with a voxel dimension of 9 μm and reconstructed using a CAD software program and a reverse engineering program (Mimics Medical 20.0; Materialize NV and Geomagic Studio 12.0; Geomagic Inc). The external and internal contours of the tooth, alveolar bone (cortical and spongious bone), 0.3-mm-thick periodontal ligament, dentin, and pulp contours were outlined and assembled. A 3-D numerical model of the intact mandibular molar was constructed by assembling all the individual elements.

### Endocrown designs

**Fig. 1:**
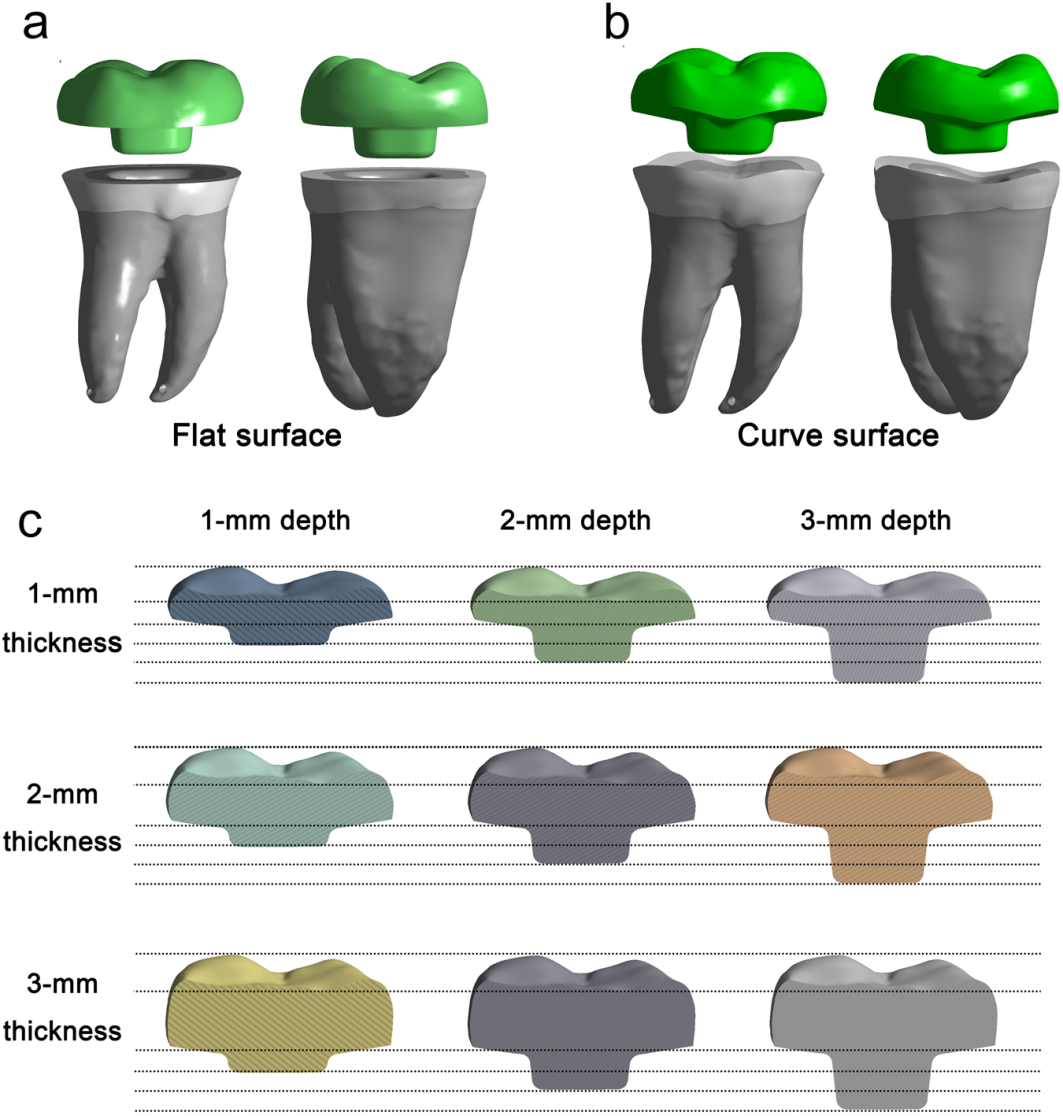
Schematic illustration of mandibular molar with endocrown restorations. Two types of butt margin of endocrown with 2-mm occlusal thickness and 2-mm pulp chamber extension depth were devised: flat surface of butt margin (**a**) and curve surface of butt margin (**b**). Nine types of endocrown restoration with 1-mm, 2-mm, or 3-mm pulp chamber extension depth in combination with 1-mm, 2-mm, or 3-mm occlusal thickness were devised by a finite element analysis software (FEA, ANSYS, v18.0; Swanson Analysis Inc) **c**

**Fig. 2:**
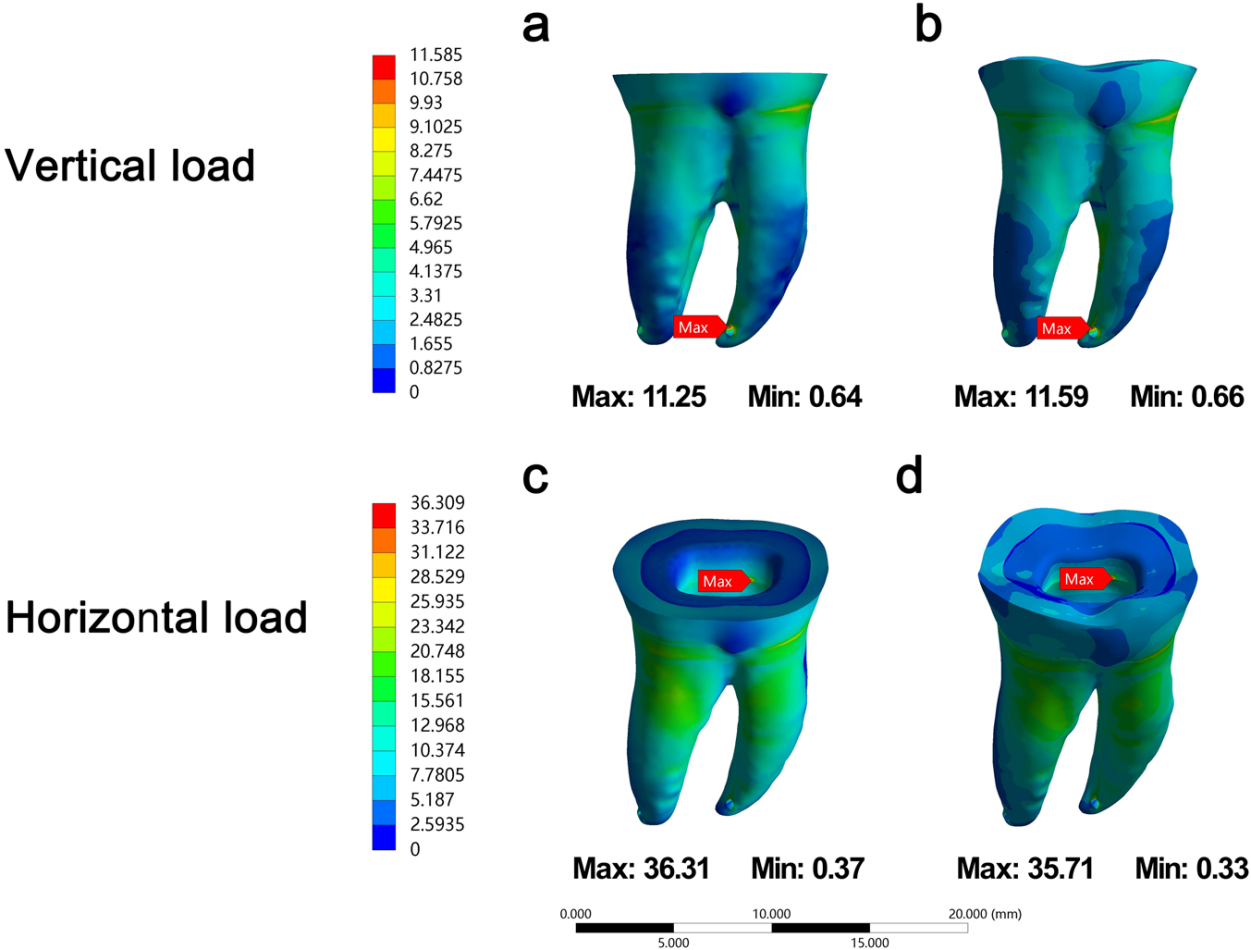
The von Mises stress (MPa) distribution in teeth tissue in flat surface (**a, c**) and curve surface (**b, d**) butt margin models under vertical load (**a, b**) and horizontal load (**c, d**). The color scale with red to blue colors corresponds to high to low-stress areas

To compare the stress distribution of endocrown restorations with two types of butt margin, 2 finite element analysis models of endocrown restoration were generated (Fig. 1a, b). (1) Flat surface of butt margin: The coronal tooth structure of the numerical model of mandibular molar was removed perpendicular to the root long axis approximately 2 mm above cemento-enamel junction (CEJ) (Fig. 1a); (2) Curve surface of butt margin: The numerical model of mandibular molar was cleaned following the occlusal morphology above 2 mm level from CEJ (Fig. 1b). Two models of endocrown restoration were designed 2-mm occlusal thickness and 2-mm pulp chamber extension depth [20], and the axial walls presented an internal taper of 12° and smooth internal transitions [12, 13]. According to the results of stress distributions on two types of butt margin, we selected curve surface of butt margin to further investigate stress distributions on endocrowns with 1-mm, 2-mm or 3-mm pulp chamber extension depth and 1-mm, 2-mm or 3-mm occlusal thickness together, and 9 FEA models were generated (Fig. 1c).

### Finite element analysis (FEA)

The constructed models simulated endocrown restoration of mandibular molar after root canal therapy. The pulp in root canal was replaced by gutta-percha, and a traditional endodontic cavity was filled with smart dentin resin (SDR) and endocrown restoration. The mechanical properties of materials, teeth tissue, and bone (Elastic modulus and Poisson’s ratio) were determined from published values. As for restoration material, lithium disilicate ceramic (IPS e.max CAD; Ivoclar Vivadent, Liechtenstein) was considered as the optimal restorative material for endocrown because of highly adhesive and excellent mechanical properties, and restorations were cemented using Mulitilink N (Ivoclar Vivadent) (Table 1). The luting cement between endocrown restorations and teeth was limited to 100-μm thickness. The models were imported into a finite element analysis software (FEA, ANSYS, v18.0; Swanson Analysis Inc.). All structures were assumed to be linearly elastic, isotropic and homogeneously distributed. Nodal displacements on the surfaces of the models were constrained in all directions. The interconnection types have been set to “bonded”. “Patch conforming method” was preferred for meshing and quadratic tetrahedral elements were used. The element size of spongious and cortical bone were defined 0.8, and other bodies was set 0.3. The number of elements and nodes of each model were generated in Table 2. A 10.6 mm-diameter of cylinder load indenter was generated by boolean operation for subtracting the restoration and simulated the full occlusal load. A vertical load of 200 N was applied to load indenter and a horizontal load of 200 N was applied at buccal direction. The two loads were applied on a solid food (apple pulp) modeled on the occlusal surface of restorations according to Ausiello et al. study [21] (Fig. 1a, b). The von Mises stress (VMS) results were obtained for all the models. For flat surface and curve surface of butt margin models, the volumes and adhesive area of enamel and dentin were calculated by using FEA software program.

**Table 1:** Material properties in the FEA models [20, 30, 31]

Material	Elastic modulus (MPa)	Poisson’s ratio
Enamel	84,100.0	0.33
Dentin	18,600.0	0.31
Periodontal ligament	68.9	0.45
Cortical bone	13,700.0	0.30
Spongious bone	1370.0	0.30
Gutta-percha	140.0	0.45
SDR	12,600.0	0.24
IPS e.max CAD	102,700.0	0.22
Multilink N	7000.0	0.30
Apple pulp	3410.0	0.10

**Table 2:** Element numbers and node numbers for the models in this study

Model		Elements	Nodes
Occlusal thickness (mm)	Pulp chamber extension depth (mm)		
2	2	458,184^a^	676,344^a^
1	1	431,629	640,774
1	2	431,011	639,878
1	3	431,310	640,467
2	1	460,153	679,642
2	2	458,312	677,147
2	3	458,898	678,103
3	1	487,167	716,449
3	2	485,926	714,819
3	3	486,381	715,597

^a^The endocrown mode with flat surface of butt margin, and the others are curve surface of butt margin

## Results

### Stress distribution of two butt margin forms of the endocrown

Stress distribution on the prepared teeth was approximately similar between flat surface and curve surface of butt margin regardless of loading direction (Fig. 2). The volume and adhesive area of the remained enamel and dentin in two butt margin models were showed in Table 3. Curve surface of butt margin offered more adhesive area of enamel and remained more volume of enamel than flat surface of butt margin. The volume of the remained dentin in two butt margin models was similar, and adhesive area of dentin in flat surface showed slightly more than that in curve surface.

**Table 3:** Volume and adhesive area of the remained enamel and dentin in two types of butt margin models in the preparation of endocrown restoration with 2-mm occlusal thickness and 2-mm pulp chamber extension depth

Butt margin form	Adhesive area (mm^2^)		Volume (mm^3^)	
	Enamel	Dentin	Enamel	Dentin
Flat surface	35.172	64.017	36.370	507.720
Curve surface	42.002	60.595	52.208	509.330

### Stress distribution of nine models under the vertical load

**Fig. 3:**
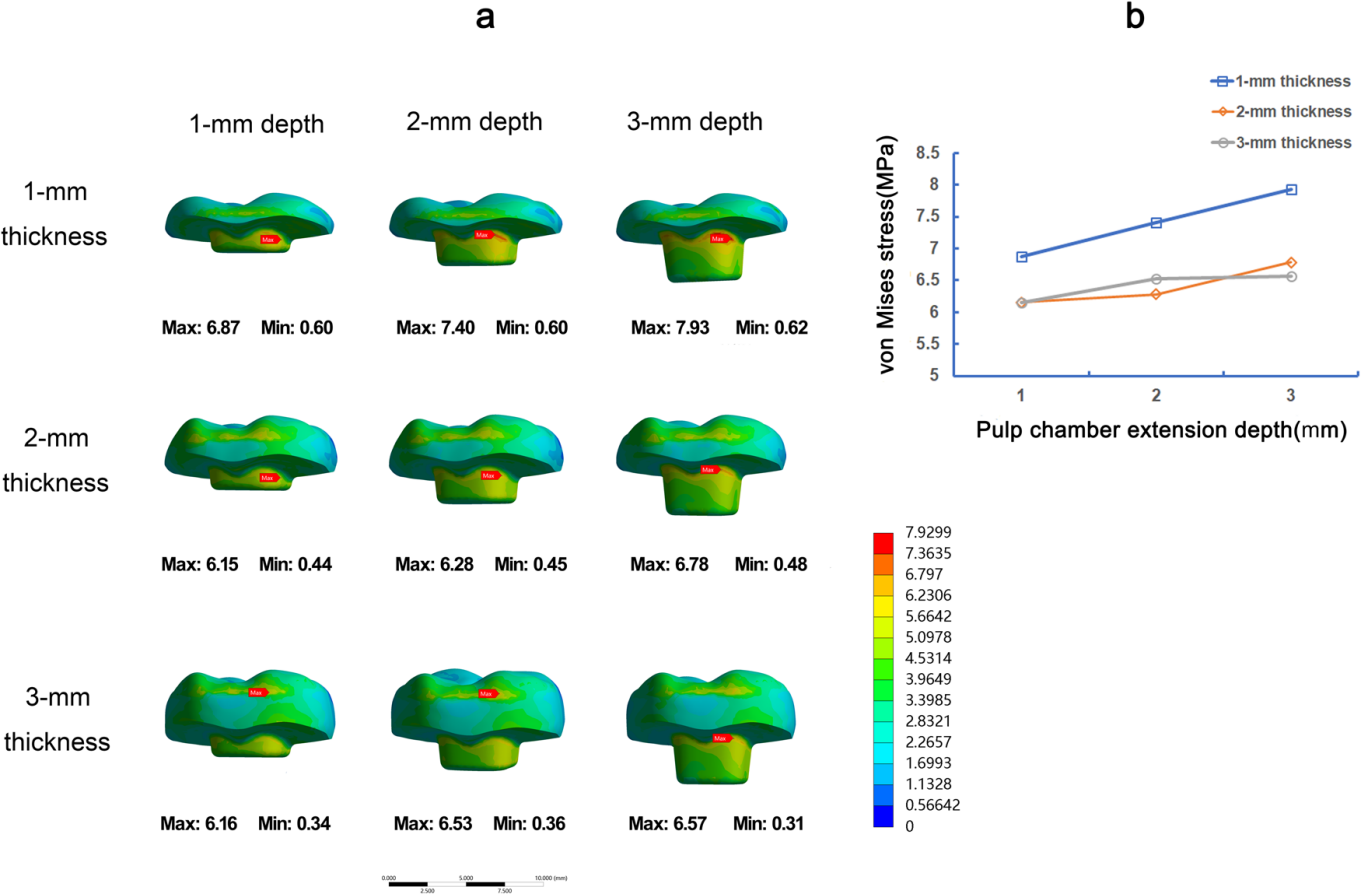
The von Mises stress (MPa) distribution on endocrown restorations in 9 FEA models under vertical load. Stress distribution plots of nine endocrown restorations with 1-mm, 2-mm, or 3-mm pulp chamber extension depth in combination with 1-mm, 2-mm, or 3-mm occlusal thickness (**a**). The color scale with red to blue color corresponds to high to low-stress areas. The maximum VMS presented on endocrown restorations with the different occlusal thickness and pulp chamber extension depth (**b**)

**Fig. 4:**
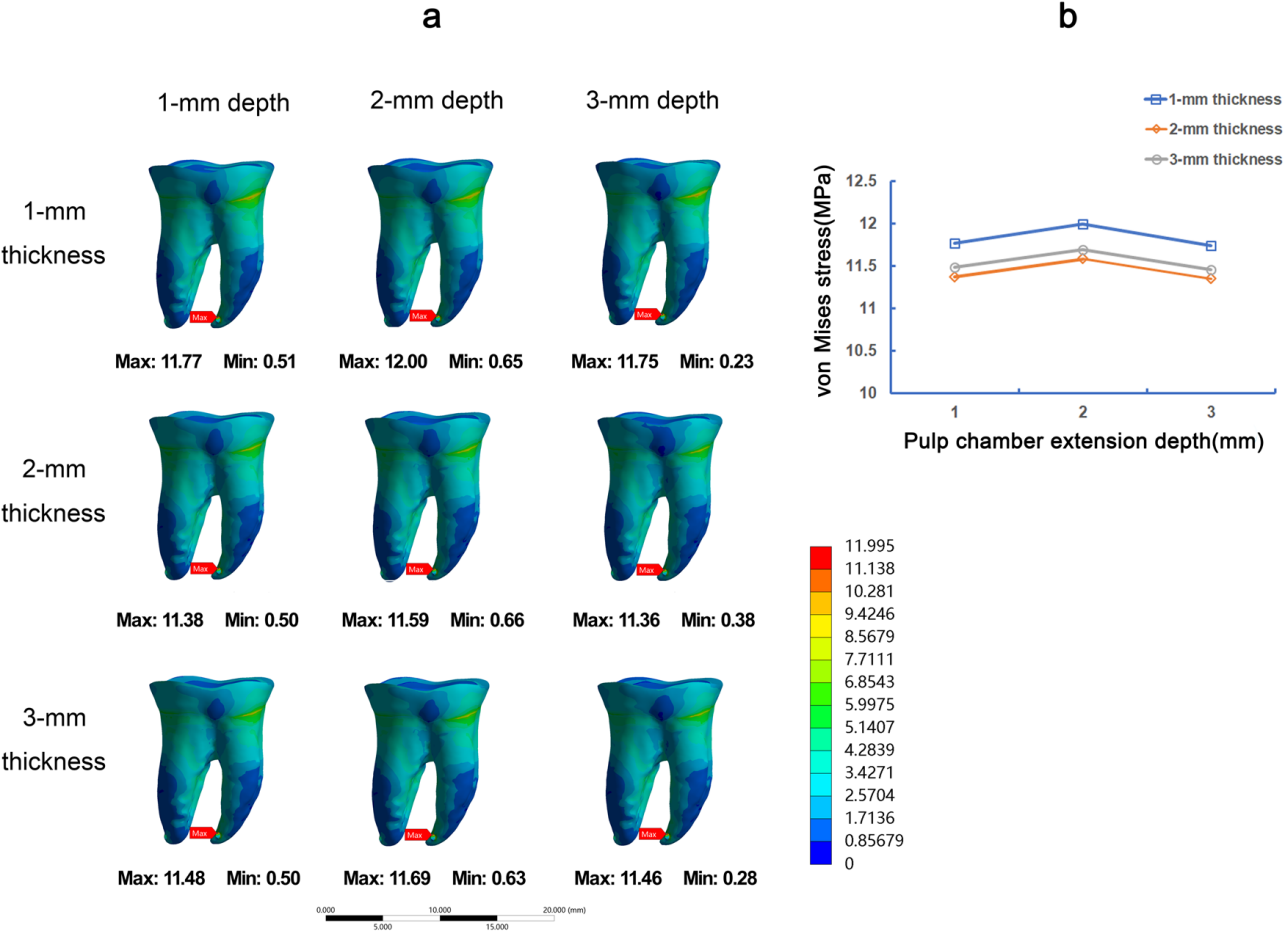
The von Mises stress (MPa) distribution on teeth tissue in 9 FEA models under vertical load. Stress distribution plots of teeth tissue by endocrown restorations with 1-mm, 2-mm, or 3-mm pulp chamber extension depth in combination with 1-mm, 2-mm, or 3-mm occlusal thickness (**a**). The color scale with red to blue color corresponds to high to low-stress areas. The maximum VMS presented on teeth tissue by endocrown restoration with the different occlusal thickness and pulp chamber extension depth (**b)**

**Fig. 5:**
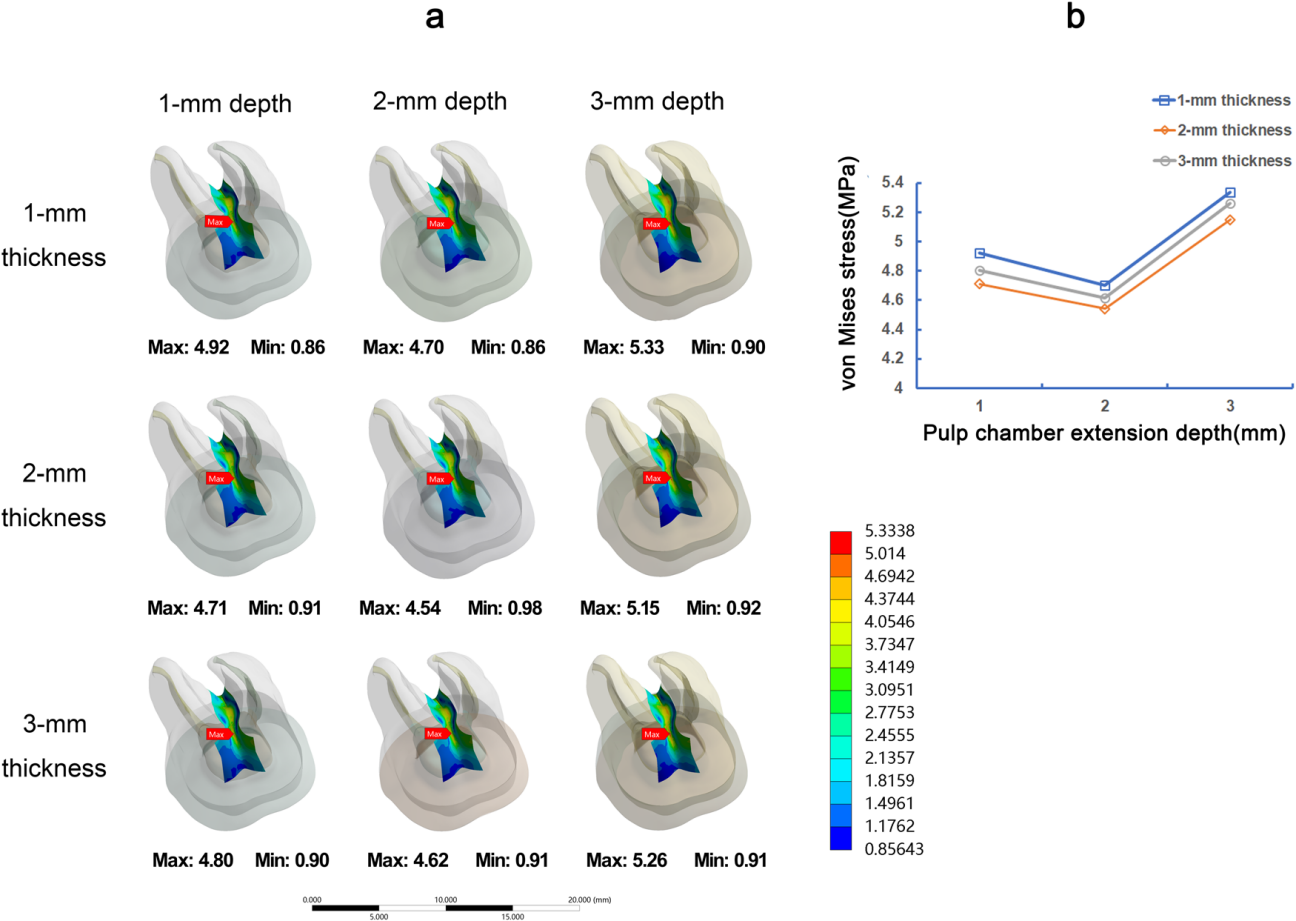
The von Mises stress (MPa) distribution in root furcations in 9 FEA models under vertical load. Stress distribution plots of root furcations of teeth by endocrown restorations with 1-mm, 2-mm, or 3-mm pulp chamber extension depth in combination with 1-mm, 2-mm, or 3-mm occlusal thickness (**a**). The color scale with red to blue color corresponds to high to low-stress areas. The maximum VMS presented on root furcations with the different occlusal thickness and pulp chamber extension depth (**b**)

Under the vertical load, the stress values on the restoration and dental tissue in 1-mm occlusal thickness were generally greater than 2-mm and 3-mm occlusal thickness (Figs. 3 and 4). The stress on restorations gradually rose with the increase of the pulp chamber depth (Fig. 3). The stress distribution on teeth tissue was about the same in 1-mm, 2-mm, and 3-mm pulp chamber extension depth, and 2-mm extension depth showed the slight high of VMS (Fig. 4). Furthermore, in root furcations of endocrown restoration, 2-mm pulp chamber extension depth showed the lowest VMS, and 3-mm extension depth showed the largest VMS (Fig. 5).

### Stress distribution of 9 models under the horizontal load

**Fig. 6:**
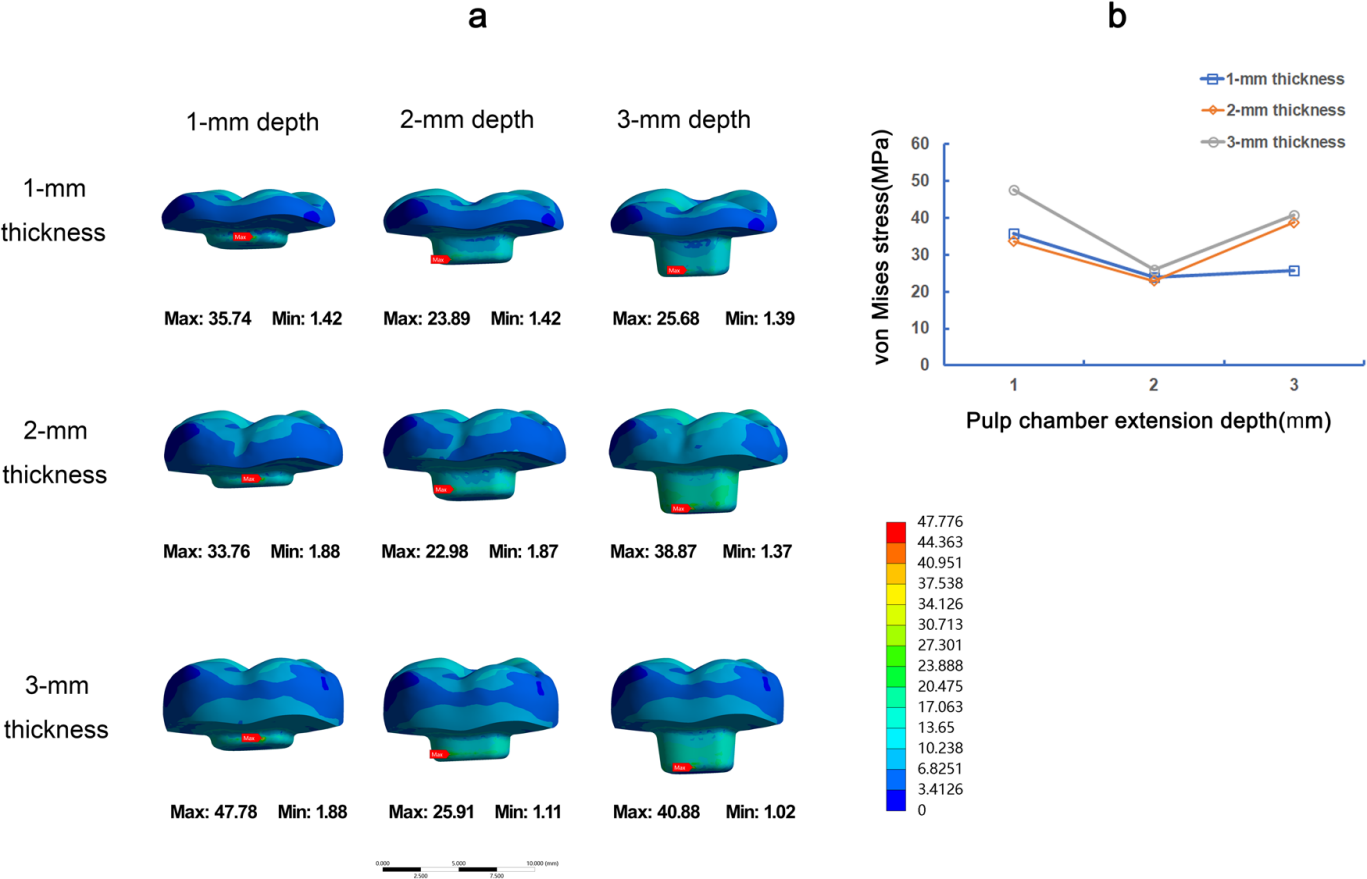
The von Mises stress (MPa) distribution on endocrown restorations in 9 FEA models under horizontal load. Stress distribution plots of nine endocrown restorations with 1-mm, 2-mm, or 3-mm pulp chamber extension depth in combination with 1-mm, 2-mm, or 3-mm occlusal thickness (**a**). The color scale with red to blue color corresponds to high to low-stress areas. The maximum VMS presented on endocrown restorations with the different occlusal thickness and pulp chamber extension depth (**b**)

**Fig. 7:**
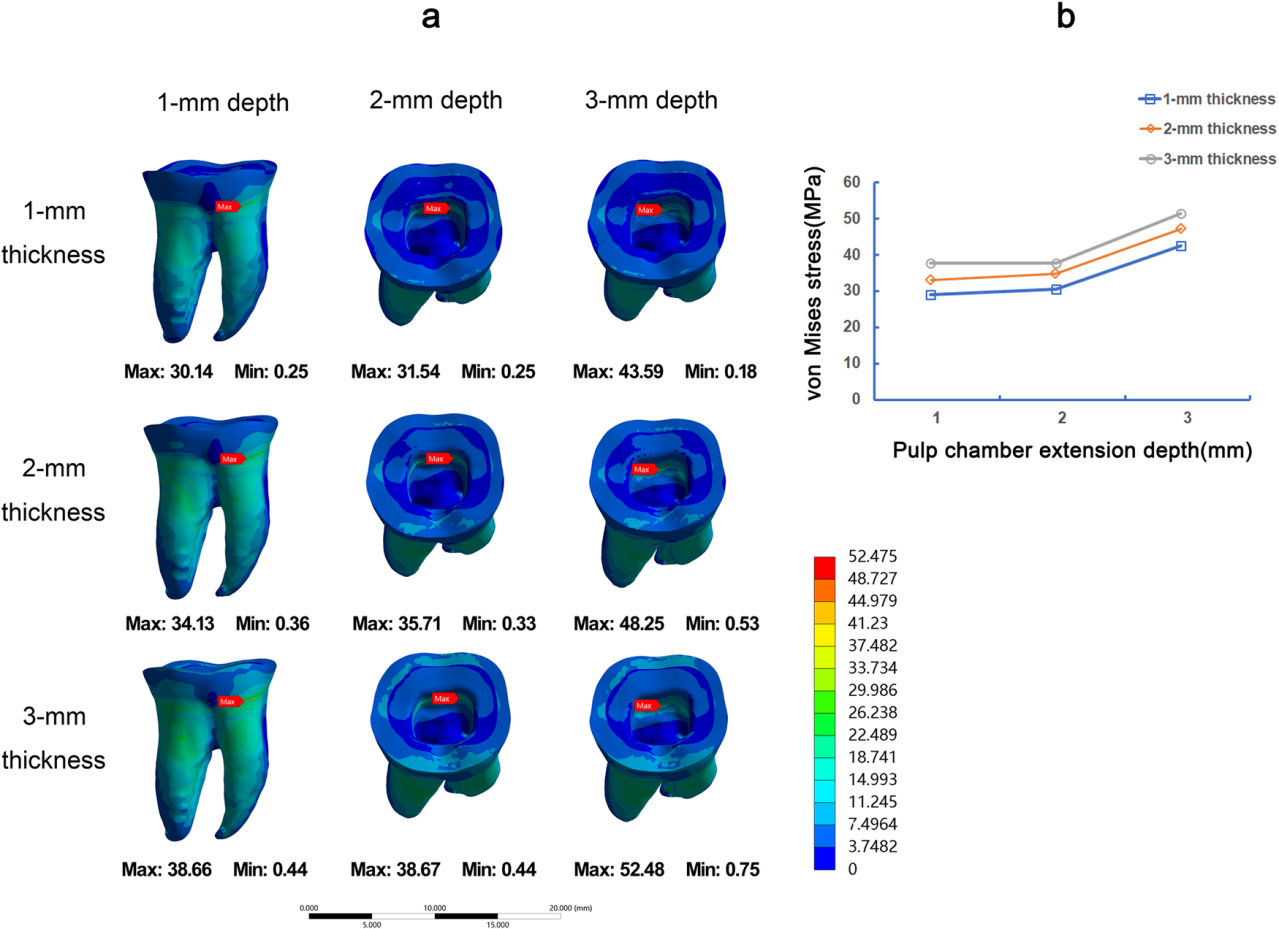
The von Mises stress (MPa) distribution on teeth tissue in 9 FEA models under horizontal load. Stress distribution plots of teeth tissue by endocrown restorations with 1-mm, 2-mm, or 3-mm pulp chamber extension depth in combination with 1-mm, 2-mm, or 3-mm occlusal thickness (**a**). The color scale with red to blue color corresponds to high to low-stress areas. The maximum VMS presented on teeth tissue by endocrown restoration with the different occlusal thickness and pulp chamber extension depth (**b**)

The stress distribution was obviously higher under the horizontal load than under the vertical load at the same 200 N load on endocrown restoration. Under horizontal load, the stress values on the restoration showed an irregular change. By and large, 1-mm extension depth of endocrown showed the largest VMS and 2-mm extension depth of endocrown showed the lowest VMS (Fig. 6). In the stress distribution on teeth tissue, 3-mm occlusal thickness of endocrown was ranked the highest, followed by 2-mm and 1-mm occlusal thickness (Fig. 7). In the evaluation of the different pulp chamber depth, the VMS of 3-mm extension depth was the highest on teeth tissue and the 1-mm and 2-mm extension depth were parallel (Fig. 7). The position of the Max VMS showed variable at 9 types of endocrown models under horizontal load. The stress peak in 1-mm extension depth was concentrated in the cervical region of outside of root, while the stress peaks in 2-mm and 3-mm extension depth were accumulated on the junction of lateral wall and bottom in pulp chamber cavity (Fig. 7).

## Discussion

The three factors: the form of butt margin, occlusal thickness, and pulp chamber extension depth were investigated stress distribution of mandibular molar ETT with endocrown restoration in this study. The different stress values were generated between three restoration thickness and three pulp chamber extensions depth, and the null hypothesis of this comparation was rejected. Stress values between flat surface and curve surface of butt margin were similar, and the null hypothesis was accepted.

Endocrown is an alternative for ETT with serious defects, vertical bite height loss or inadequate clinical crown length. Accurate tooth preparation strategy about how to design pulp chamber of endocrown restoration is still a clinical dilemma for dentists. The occlusal thickness of endocrown depends on the removal amount of tooth tissue in preparation, except for lesion of tooth. A few researchers mostly considered the influence of pulp chamber extension depth of endocrown, but the design of pulp chamber extensions depth might be affected by occlusal thickness, especially under horizontal load due to lever principal. Therefore, pulp chamber extension depth and occlusal thickness should be together considered to study stress distribution on endocrown restoration. In design, we at first need to consider the factor of butt margin. In preparation of conventional full crown restoration, an evenly removal is generally required according to occlusal anatomy. Similarly, due to occlusal anatomy, curve surface of butt margin was compared with flat surface of butt margin in stress distribution of endocrown restoration by finite element analysis. FEA has been widely applied in dental biomechanical studies to examine the stresses generated on teeth tissue and predict clinical performance of restorations [12, 14, 19, 22, 23]. Though flat surface and curve surface of butt margin showed similar VMS in endocrown restoration, curve surface of butt margin preserved more enamel and offered more adhesive area, which benefited for bonding of endocrown restoration. These results indicated that curve surface of butt margin may be preferable in endocrown restoration.

In view of the results of curve surface and flat surface of butt margin, we selected curve surface of butt margin to complete the nine types of FEA endocrown model. Endocrown consists of the core-portion inside the pulp chamber and occlusal-portion covering the crown. As a one-piece “monoblock”, some studies evaluated the change of occlusal thickness and pulp chamber extension depth under the invariable height of endocrown restorations [10, 18, 19]. In the study by Tribst et al., the three same height of FEA endocrown models under the different remnant amount of tooth tissue (1.5, 3 and 4.5 mm height of remnant tooth) showed the greater the dental crown remnant and the thinner the restoration, the higher the stress concentration on the restoration [19]. In an in vitro study by Taha et al., endocrowns restoration with 3.5 mm of occlusal thickness had higher fracture resistance mean values than those with 2 mm of occlusal thickness under the same height of restoration [10]. Haralur et al. considered that the increased occlusal thickness significantly improved the fracture strength of molar endocrowns [18]. The studies based on the invariable restoration height and flat surface of butt margin of endocrowns. Our nine FEA endocrown models, based on the curve surface of butt margin and the same 2 mm height dental crown remnant, also showed that 1-mm occlusal thickness had higher stress distribution on both restoration and teeth tissue than 2-mm and 3-mm occlusal thickness at any a pulp chamber extension depth under the vertical load. At the horizontal load in the 9 FEA models, 1-mm and 2-mm occlusal thickness generated lower stress on tooth tissue than 3-mm occlusal thickness, and 2-mm occlusal thickness formed the relatively low stress on restorations. As a result, the results indicated that 2-mm occlusal thickness was desirable in endocrown restorations.

Unlike occlusal thickness, pulp chamber extensions depth may be a relatively controlled factor in endocrown restorations. Pulp chamber of ETT was generally filled with composite resin close to elastic modulus of dentin so as to direct extension depth of restorations. At present, there was no agreement in reports on pulp chamber extension depth in preparation of endocrowns. In the study by Kuijper et al., 0, 2 and 4 mm extension depth in pulp chamber did not significantly influence the load to fracture of glass-ceramic endocrown restorations of the consistent residual dental tissue after extensive thermomechanical aging [24]. Dartora et al. set up three CAD/CAM molar endocrown restorations with 1, 3, and 5-mm pulp chamber extension depths, and found that more pulp chamber extension depth provided better mechanical performance in the axial load [14]. Though the deeper the pulp chamber extension for an endocrown and the greater the surface area for adhesive retention, the better the transmission of masticatory forces to the root, 5-mm extension depths might destroy the pulp chamber floor of mandibular molar, according to the anatomical data of mandibular molar pulp chamber height [25, 26]. In another study by Hayes et al., more extension depths in pulp chamber increased the probability of catastrophic fracture in the load of 45° angle to the axis of the tooth in evaluation of 2, 3, and 4-mm pulp chamber extension depths and thereby the endocrown pulp chamber extension should not be deeper [16]. Furthermore, the other two researches also showed that 2-mm depth was acceptable [8, 17]. According to the data in these studies, we did not devise more deep pulp chamber extension to set up FEA endocrown models, and the effect of 1, 2, and 3-mm extension depth on the stress distribution of endocrown was investigated in our FEA models.

Our studies indicated that 3-mm extension depth generated higher stress values on teeth tissue than 1-mm and 2-mm extension depth under horizontal load. At the load of a 45° angle to the long axis of the tooth in Hayes et al. study, the mean of failure force of 3-mm pulp chamber extension depth was 762.8 N and 92% nonrestorable failures, but 843.4 N and 66% in 2-mm extension depth [16]. These results indicated that the deeper extension of pulp chamber caused higher stress on surrounding structures, and may increase the risk for fracture. However, in a premolar with endocrown restoration at 2 and 3 mm extension depth, two intracoronal cavity depths had no correlation with fracture resistance and microleakage at a vertical load [27]. Therefore, the different extension depth might show the different stress distribution results, with reference to the load direction, restoration materials, and tooth types, etc. Furthermore, endocrown restoration transmits stress to root furcations due to extension in pulp chamber under the vertical load [12, 22, 23]. We further evaluated the stress in root furcations under axial load and found that 2-mm extension depth of endocrown showed the lowest VMS, and 3-mm extension depth of endocrown showed the largest VMS. As a result, the deeper pulp chamber extension was not recommended, especially for cases where pulp chamber floor was weak and complicated by furcation involvement.

The study synthetically analyzed stress distributions on endocrown restorations with the variation in both pulp chamber extension depth and occlusal thickness. We found that both factors could influence stress distributions of restorations and teeth tissue, and the variation of stress distribution on restorations changing as the pulp chamber extension depth of 1-mm occlusal thickness were different from 2-mm and 3-mm occlusal thickness. Therefore, it was necessary to devise different occlusal thicknesses when we evaluated the influence of pulp chamber extension depth. One limitation of this study was the choice of a common restorative material lithium disilicate ceramic, because restorative material is one of the important factors that might affect restorative performance [28, 29], and thereby the different types of restorative materials needed to be further investigated. In view of the stress distribution pattern of endocrown with different pulp chamber extension depth and occlusal thickness based on the Young’s modulus and Poisson’s ratio of the materials, further in vitro experiment of fracture resistance and clinical trials are required to validate the results of this FEA study.

## Conclusion

The results of this study will help provide a clinical basis for clinicians to restore endodontically treated teeth by EMAX endocrowns. Within the limitations of this FEA study of EMAX endocrown restoration, the following conclusions were drawn:

1. Though stress distribution of curve surface and flat surface of butt margin were similar, the former was preferable in EMAX endocrown restoration due to the more adhesive surface and preservation of enamel.
2. The endocrown with 2-mm occlusal thickness and 2-mm pulp chamber extension depth showed advantage on stress distribution on the EMAX restoration and tooth tissue compare to the other types of FEA models regardless of load direction.

## Supplementary information

Supplementary Material

Supplementary Figure 1

Supplementary Figure 2
