# Supplementary figures and images for: The synergetic effect of pulp chamber extension depth and occlusal thickness on stress distribution of molar endocrowns: a 3-dimensional finite element analysis
Source: J Mater Sci Mater Med. 2022 Jun 20;33(7):56. doi: 10.1007/s10856-022-06677-0 (PMC9209353; doi:10.1007/s10856-022-06677-0)

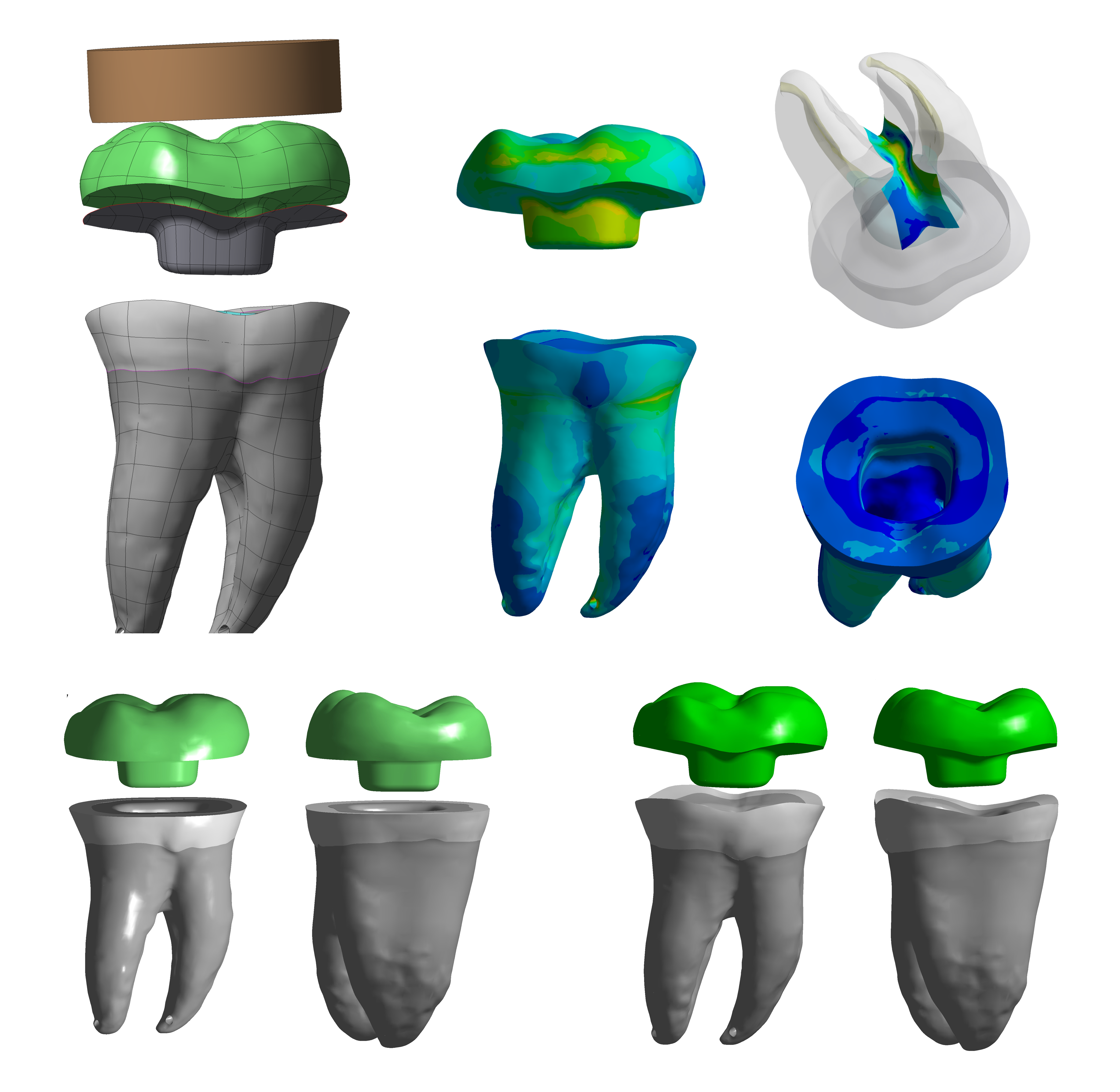

Supplement: Supplementary file 1 — Supplementary Material [file 10856_2022_6677_MOESM1_ESM.tif]
